# Increased TNF-α, IL-6 and decreased IL-1β immunohistochemical expression by the stromal spindle-shaped cells in the central giant cell granuloma of the jaws

**DOI:** 10.4317/medoral.17205

**Published:** 2011-12-06

**Authors:** Panagiota Papanicolaou, Evanthia Chrysomali, Evangelia Stylogianni, Catherine Donta, Dimitris Vlachodimitropoulos

**Affiliations:** 1 DDS, McS, DrDent. Clinical Assistant, Department of Oral and Maxillofacial Surgery, School of Dentistry, University of Athens, Athens, Greece; 2 DDS , DrDent. Ass istant Profess or, Department of Oral Pathology, School of Dentistry,University of Athens, Athens, Greece; 3 DDS , DrDent. Ass istant Profess or, Department of Oral and Maxillofacial Surgery, School of Dentistry,University of Athens, Athens, Greece; 4 DDS , DrDent. Ass istant Profess or, Department of Oral Diagnosis and Radiology, School of Dentistry,University of Athens, Athens, Greece; 5 MD , Dr.M ed. Ass istant Profess or, Laboratory of Forensic Medicine and Toxicology, Medical School, University of Athens, Athens, Greece

## Abstract

Objectives: the exp ress ion of the osteoclastogenic cytokines TNF-α, IL-6 and IL-1β were immunohistochemically evaluated in periph eral (PGCG) and central (CGCG) giant cell granulomas of the jaws in order to determine diff erences between these two lesions and between the two distinct tumor cell populations (multinucleated giant cells, MGCs and stromal sp indle-sh aped cells). Study Design: Paraffin-embedd ed tiss ue sections from 40 PGCG and 40 CGCG were immunohistochemically stained using antibodies against TNF-α, IL-6 and IL-1β. The percentage of positively stained cells and the staining intensity were ass ess ed to provide a combined immunoreactivity score value. Results: TNF-α, IL-6 and IL-1β were exp ress ed in all lesions. The CGCG compared to the PGCG sh owed significantly increased exp ress ion of TNF-α and IL-6 and decreased exp ress ion of IL-1β by the sp indle-sh aped cells and increased exp ress ion of IL-1β by the MGCs. The MGCs demonstrated in comparison to the stromal sp indlesh aped cells significantly increased exp ress ion of all three cytokines in both PGCG and CGCG. Conclusions: The proinflammatory cytokines TNF-α, IL-6 and IL-1β seem to be involved in the growth process of PGCG and CGCG of the jaws. A poss ible alteration in the sy nthesis or/and activity of these cytokines by the stromal sp indle cells in the CGCGs may enhance osteolys is through the stimulation of osteoclast progenitor cells, given the fact that the intraoss eous lesions cause bone resorption.

** Key words:** Giant cell granuloma, giant cell tumor, multinucleated giant cells, jaw, TNF-alpha, IL-6, IL-1beta, immunohistochemistry.

## Introduction

The central giant cell granuloma (CGCG) of the jaws represents a non-neoplastic and localized benign but sometimes aggressive osteolytic proliferation (1). Τhis entity most commonly occurs in the mandible as an expansile radiolucency, shows variable clinical behaviour and a subset of lesions may exhibit locally aggressive growth pattern with rapid tumour enlargement associated with teeth displacement, root resorption or bone cortical perforation (2-4). The origin of CGCG is uncertain. Local trauma, inflammation, intraosseous bleeding and genetic abnormalities have been regarded as possible causes, but a unique explanation has not gained a wide acceptance (5).

The peripheral giant cell granuloma (PGCG) is an extraosseous giant cell lesion. In the oral cavity this lesion presents as a sessile or pedunculated purple-brown exophytic mass, located on the gingival or the alveolar mucosa (4). In some cases of PGCG a slight superficial (cupping) erosion of the adjacent bone can be seen radiologically (6). The exact aetiology of PGCGs still remains unclear. Developmental or/and inflammatory reactions in the periodontal ligament or the periosteum have been proposed to be involved (4,6). Local irritation factors such as poor dental restorations, unstable dental prosthesis, dental extractions, plaque and calculus accumulation and food retention seem to play a significant role in the development of a PGCG (7).

The two lesions (CGCG and PGCG) demonstrate identical histopathologic features (4). They are both characterized by numerous multinucleated giant cells in a fibroblastic vascularized stroma with ovoid to spindle-shaped cells which are thought to compose a heterogenous population of macrophage and fibroblastic-like cells (8).

The origin of the multinucleated giant cells (MGCs) has been a matter of considerable interest. These cells are considered to be formed from the fusion of monocyte/macrophage precursors differentiated into osteoclasts under the influence of cytokines (8-10). The mononuclear cell component of the GCGs consists of a population of macrophage-like cells, which appears to include a subset of osteoclast precursors, and a proliferating spindle-shaped stromal cell population which has the capacity to differentiate along fibroblast/osteoblast lines. The fibroblast/osteoblast-like cells in GCGs of the jaw expressing the receptor activator of NF-κB ligand - RANKL, which is a primary mediator of osteoclast differentiation, activation, and survival, are responsible for inducing the formation of osteoclast-like MGCs from monocytes/macrophages found in these lesions (11,12).

Many osteotropic hormones and cytokines have direct or indirect stimulatory and antagonistic effects on the development of the osteoclasts. TNF-α is a multifunctional cytokine released by activated monocytes, macrophages and T lymphocytes and contributes to immune responses, regulating growth, differentiation, and further production of other cytokines, inflammatory mediators and enzymes. TNF-α is a potent bone resorption inducer that stimulates osteoclast differentiation and activation. The proinflammatory cytokines IL-6 and IL-1β, products of stromal cells and monocytes, stimulate in association with TNF-α osteoclast differentiation and activation in a synergistic fashion. These cytokines not only regulate osteoclastogenesis by stromal cells, but also act directly on osteoclasts and their precursors (11,13,14). 

The role of TNF-α, IL-6 and IL-1β in the pathogenesis of osteolytic lesions and diseases with pathologic bone resorption has been proved (11). These osteoclastogenic cytokines have been investigated by using various methods in giant cell tumors (GCTs) of long bones in several studies in vivo and in vitro (8,15-17). The expression of TNF-α has also been studied in patients with giant cell lesions of the jaws (18,19).

In the present study, the TNF-α, IL-6 and IL-1β expression were immunohistochemically evaluated in peripheral and central giant cell granulomas of the jaws, in order to determine possible differences between these two entities and between multinucleated giant cells and stromal spindle-shaped cells. To our knowledge, there are no studies in the English literature concerning the in situ comparative immunohistochemical expression of this triad of cytokines between the PGCG and CGCG of the jaws.

## Material and methods 

 Study group

In this study, files of patients with a definite diagnosis of PGCG and CGCG from the Department of Oral Pathology and Medicine, Faculty of Dentistry, University of Athens were revised, the diagnosis in each case having been made on the basis of clinical, radiologic and histologic findings. Formalinfixed and paraffin-embedded - tissue samples of all the cases were retrieved. All specimens were obtained from surgical excision of the lesions and had been fixed in 10% buffered formalin. Assessment of hematoxylin and eosin-stained sections of all cases together with evaluation of clinical and radiologic data too were done so as the diagnosis of the lesions to be confirmed. Forty (n=40) cases of PGCGs and forty (n=40) cases of CGCGs were selected. The selection criteria included the presence in the patients’ records of detailed clinical information (age, gender, anatomic location, clinical features, signs and symptoms at presentation) as well as radiographs (intraoral radiographies for the cases of PGCGs, panoramic radiographies and/or CT scans for the cases of CGCGs). In all the cases of CGCGs, laboratory tests values for serum calcium and phosphorus concentrations, alkaline phosphatase activity and parathyroid hormone level were also available. In all patients these parameters were within the reference ranges, excluding the occurrence of other diseases which could compromise the final diagnosis of CGCG.

Most patients were females (55% of cases with PGCG and 70% of cases with CGCG). The mean age of patients with PGCG and CGCG was 49.2 years and 45 years respectively. Both PGCG and CGCG showed a predilection for the anterior region of the mandible (75% of PGCG and 67% of CGCG). PGCGs mainly appeared as red pedunculated lesions with smooth surface and rubbery consistency (59%), while in 34% of the cases a superficial, cup-shaped radiolucency was seen radiographically. The most common clinical finding of CGCGs was bony expansion of the jaw (75%), while in more aggressive lesions cortical perforation, tooth displacement and, rarely, pain and paresthesia were also observed. In radiographic examination, a unilocular or multilocular radiolucency was observed.

 Immunohistochemistry

The immunohistochemical staining was performed on 3-5μm thick paraffin-embedded tissue sections. Sections were deparaffinized in xylene, hydrated through graded alcohol and washed with Tris-buffered saline (TBS) for 10 min and distilled water for another 10 min. Endogenous peroxidase activity was blocked with 3% v/v H2O2 in water for 5-min. Antigen retrieval was performed for all antibodies by placement of the sections in citrate buffer and heating in microwave oven for 15 min. Sections were separately incubated overnight with the primary antibodies for TNF-α (mouse, monoclonal, HM2010, Hycult Biotech, dilution 1:10), IL-6 (goat, polyclonal, Sc1265, Santa Cruz Biotech, dilution 1:150) and IL-1β (mouse, monoclonal, Sc52012, Santa Cruz Biotech, dilution 1:400) and then incubated in the biotin-conjugated secondary antibody for 10 min. The standard strepta-vidin–biotin–peroxidase complex method was performed to bind the primary antibody with the use of a LSAB System Universal Kit (Dako) for 10 min, DAB solution was used as chromogen for 5 min and all sections were counterstained with Mayer΄s haematoxylin for 1 min and mounted. Positive tissue controls included human inflamed skin for TNF-α, glioblastoma for IL-6 and pilonidal cyst for IL-1β. For negative controls slides the antibody was omitted. 

In each section, four high-power fields were randomly selected, with a 40X magnification, and the percentage of positively stained cells (PP) of the MGCs and stromal spindle-shaped cells was assessed in each field by two observers as: 0 (<10;% stained cells), 1 (≥10%), 2 (≥25%), 3 (≥50%), and 4 (≥75%). Staining intensity (SI) was graded as: 0=no expression, 1=weak, 2=moderate and 3=strong. The immunohistochemical expression for each cytokine was evaluated by using the scoring method ImmunoReactivity Score «IRS» (20,21). According to this, the score of the percentage of stained cells (PP) for each field was multiplied by the score of the staining intensity (SI) to provide a combined immunoreactivity score value (IRS) (IRS: PP×SI). The mean of the four fields was the IRS score for the sample.

 Statistical analysis

Statistical analysis was performed by SPSS 13.0 statistical package. Because the data were conformed to abnormal distributions, the non-parametric Mann-Whitney was used. The differences were considered as statistically significant at level p=0.05.

## Results

All tumors showed similar histological features exhibiting a great number of MGCs surrounded by cell populations with oval to spindle cell morphology in a loose fibrillar connective tissue stroma with many small blood vessels. Hemosiderin (11 cases in CGCG and 7 cases in PGCG) and bone formation demonstrating immature osteoid trabeculae surrounded by numerous osteoblasts (19 and 12 cases in CGCG and PGCG respectively) were often seen. 

**Table 1:**
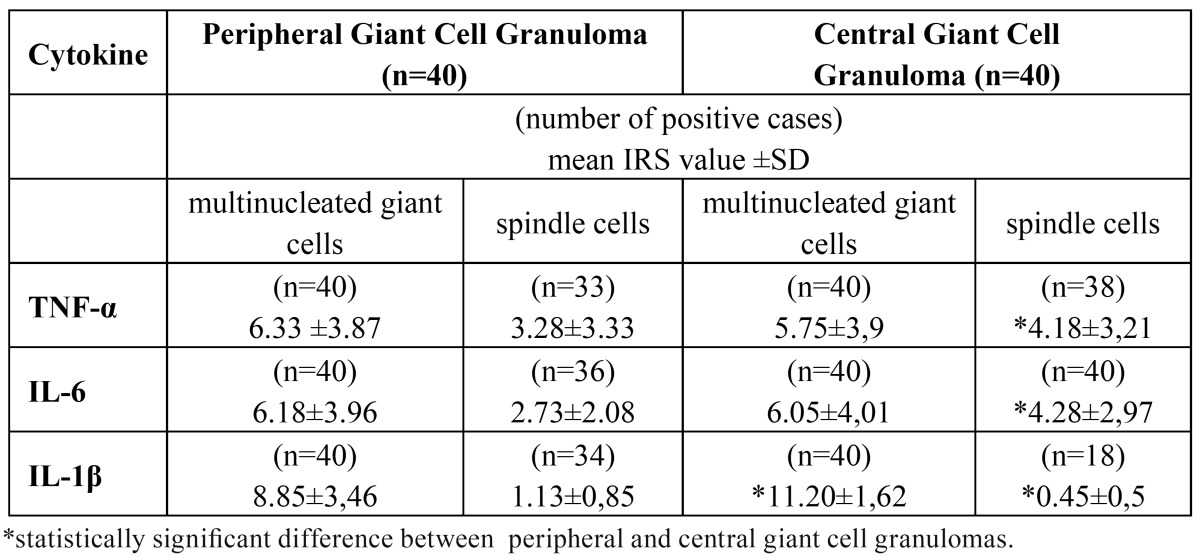
Immunoreactivity score (IRS) values for TNF-α, IL-6 and IL-1β in multinucleated giant cells and stromal spindle-shaped cells. Comparison between the peripheral and central giant cell granulomas.

**Figure 1:**
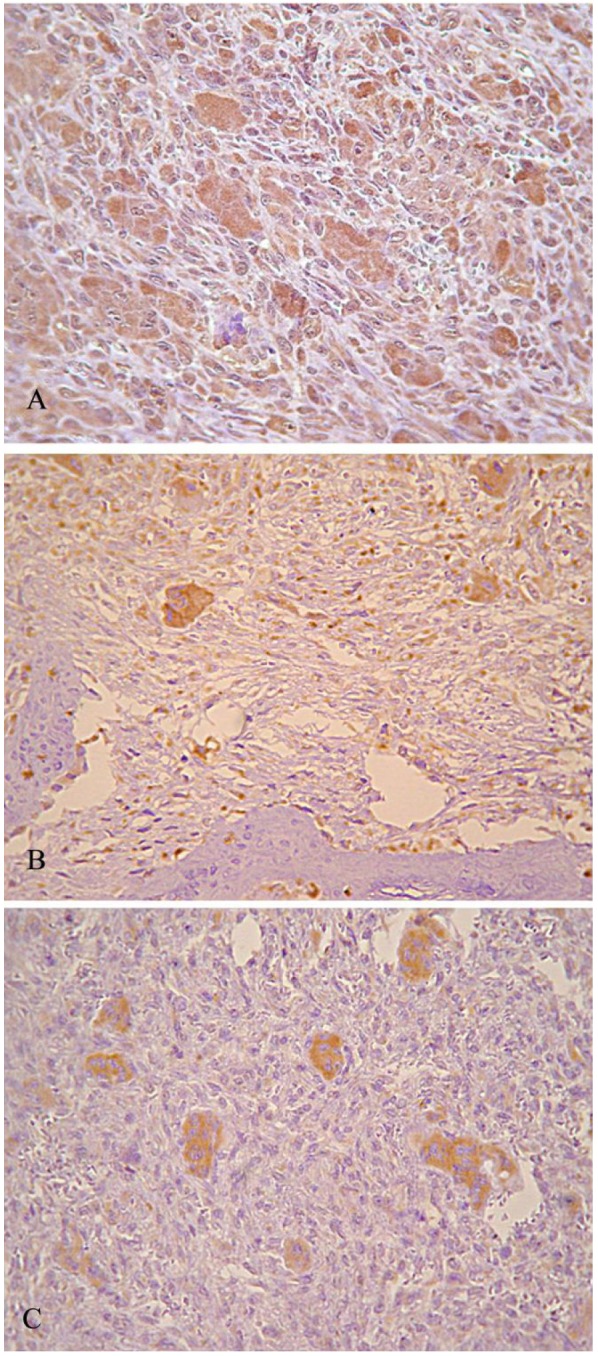
(A) TNF-α immunohistochemical exp xp ress ss ion in PGCG (Χ60). Strong staining intensity of MGCs Cs and moderate staining intensity in stromal sp sp indle-sh sh aped cells, (B) IL-6 immunohistochemical exp xp ress ss ion in CGCG (Χ60). Strong staining intensity of MGCs Cs and moderate staining intensity in stromal sp sp indle-sh sh aped cells, (C) IL-1β immunohistochemical exp xp ress ss ion in CGCG (Χ120). Strong staining intensity of MGCs Cs for IL-1β and no immunoreactivity in stromal sp sp indle-sh sh aped cells.

TNF-α, IL-6 and IL-1β were detected in all cases of PGCGs and CGCGs ( Table 1). The MGCs expressed TNF-α, IL-6 and IL-1β in all cases of PGCGs and CGCGs as cytoplasmic immunostaining (Figure 1 A,B,C). The giant cells demonstrated in comparison to the stromal spindle-shaped cells significantly increased expression of all three cytokines in both PGCG and CGCG. A variable expression for the examined cytokines was observed from the stromal spindle-shaped cells in peripheral and central lesions ( Table 1). Specifically, in 95% of the central and in 82.5% of the peripheral GCGs, the spindle cells showed expression for TNF-α (Fig. 1A). IL-6 was expressed by the stromal spindle-shaped cells in all cases of the CGCGs (Fig. 1B) and in 90% of PGCGs, while IL-1β was expressed by these cells in 85% of the peripheral and 45% of the central lesions. 

There was no statistically significant difference between PGCG and CGCG considering the expression of TNF-α and IL-6 by MGCs, in contrast to the stromal spindle-shaped cells, which in CGCGs showed a significantly increased expression of these two cytokines ( Table 1). The comparison between CGCG and PGCG considering the expression of IL-1β revealed that the CGCG showed significantly increased IL-1β expression by MGCs and decreased IL-1β expression by stromal spindle-shaped cells (Fig. 1C).

## Discussion

The osteoclastogenic cytokines TNF-α, IL-6 and IL-1β have been investigated in giant cell tumors (GCTs) of long bones. Whether the GCGs of the jaws and the GCTs of long bones are really a single pathologic process is an unanswered question. They are both characterized by the presence of MGCs , although GCTs may exhibit higher mean number of giant cells per measurement field, higher number of nuclei per giant cell, greater fractional area and relative size index and higher necrosis (22,23). CGCGs, despite their reactive nature, show higher proliferative activity (24). They are much less destructive and tend to involve a younger age group (23). GCTs show neoplastic characteristics (24). They are usually painful and fast growing (5), characterized by an unpredictable biological behavior, local aggressiveness and high recurrence rates (25). The distinction between the GCGs and the GCTs may be controversial, but their histopathological and immunohistochemical similarities seem to reflect a similar pathogenesis, considered to represent a spectrum of the same disease process (22,23).

To our knowledge this is the first study regarding simultaneously determination of these three proinflammatory cytokines by both MGCs and stromal spindle-shaped cells in PGCGs and CGCGs of the jaws. In a previous study, Amaral et al. (18) investigated the expression of TNF-α in peripheral and central giant cell lesions of the jaws and found decreased transcription of TNF-α genes in all the cases studied compared to healthy control samples. In addition, De Souza et al. (19) evaluated TNF-α expression by circulating lymphocytes and monocytes in patients with central giant cell lesions of the jaws and found increased expression of this cytokine by CD4(+) T cells and decreased frequency of TNF-α+ cells in CD68+ circulating monocytes. The authors propose that as the results of the study demonstrated an increased expression of IL-10 by monocytes and since IL-10 inhibits IL-1, IL-6 and TNF-α production by activated macrophages (24), the higher expression of IL-10 could possibly explain the observed decreased frequency of TNF-α+ cells. According to this study, it is interesting that although central giant cell lesions are localised in the jaws, they may cause significant systemic functional alterations in circulating leukocytes. Whether the central giant cell lesions may cause peripheral leukocyte activation or this activation may be caused by other factors and stimulates the formation of central giant cell lesions remains to be elucidated (19). 

The results of the present study are in agreement with those found in GCTs, which show variable expression of the TNF-α, IL-6 and IL-1 by multinucleated giant cells and stromal spindle-shaped cells (8, 15-17). The TNF-α, IL-6 and IL-1β expression in peripheral and central GCGs of the jaw indicates that these cytokines are implicated in the growth process of both extraosseous and intraosseous giant cell lesions supporting the previously stated opinion that peripheral and central GCGs share similar growth potential (26,27). These osteoclastogenic cytokines comprise a triad of factors that interact and may play a critical role in MGCs formation regulating the bone resorption (28), suggesting a possible synergistic role in the development of GCGs. Τhe MGCs exhibit functional and phenotypic characteristics of osteoclasts including tartrateresistant acid phosphatase, amino-peptidase, V-ATPase, CA II, Cathepsin K, MMP-9 and CD68, vitronectin and calcitonin receptor. The above similarities between MGCs and osteoclasts are indicative for a common histogenesis (12,21,29). Recently, the study of Amaral et al. (18) in PGCGs and CGCGs showed increased transcription of the nuclear factor of activated T cells (NFATcl), which is a master of transcription in terminal differentiation of osteoclasts. These authors proposed that the development of giant cell lesions of the jaws is possibly mediated by overexpression of NFAT in the nucleus of the MGGs. In the present study, the MGCs immunostaining for TNF-α, IL-6 and IL-1β may possibly be related to osteoclast differentiation.

The spindle-shaped cells in the mononuclear cell component of the PGCG and CGCG have been proved to represent the “proliferating compartment” considered responsible for the biologic activity of these tumors. These mesenchymal in origin cells resembling immature osteoblasts release a wide range of factors (receptor activator of NF-κB ligand - RANKL, interleukines, interferon gamma - IFN-γ, macrophage-colony stimulating factor - MCSF, granulocytemacrophage colony stimulating factor - GMCSF) that recruit monocytes/osteoclast precursors and promote their differentiation into functional osteoclasts (8,21, 26-28). In the present study, the comparison between the peripheral and central GCGs revealed that in the CGCG there was a statistically significant increased expression of TNF-α and IL-6 by the spindle cells, but not by the MGCs. TNF-α is responsible for stimulating osteoclastic bone resorption in vitro as well as in vivo (11,14). IL-6 is known to stimulate mesenchymal progenitor differentiation toward the osteoblastic lineage and is also a potent antiapoptotic agent on osteoblastic cells. The main sources of IL-6 in bone are osteoblastic cells, stromal cells and not osteoclastic cells, but the activity of IL-6 on bone is its effect on osteoclastogenesis (11).

In contrast to the increased expression of TNF-α and IL-6 by the spindle cells in our study, the CGCG showed significantly increased IL-1β expression by the MGCs and decreased IL-1β expression by the stromal spindle-shaped cells. Gamberi et al. (15) by using immunohistochemistry and real-time quantitative PCR techniques found increased expression of IL-6 in GCTs associated with higher biological aggressiveness, but without any significant differences between the two cell populations. Increased expression of TNF-α, IL-1 and IL-6 mRNA by stromal cells has been observed in GCTs by Atkins et al (16). On the other hand, immunoreactivity for IL-1β, IL-6 and TNF-α also confirmed by in situ hybridization was mainly observed in giant cells, whereas stromal cells showed scattered staining in GCTs (17).

Regezi (26) speculates that the subset of CGCGs that show a locally aggressive behaviour may develop from a reactive lesion through an epigenetic event occurring in spindle-shaped mesenchymal cells of bone resulting in escape from cell cycle controls and in expression of proteins capable of monocyte recruitment and differentiation into MGCs. Osteoclastic differentiation is controlled by complex interactions between OPG, RANK and RANKL. OPG and RANKL are synthesized by stromal cells/osteoblasts, while RANK is localized at the cell surface of mature osteoclasts and osteoclastic precursors. OPG inhibits osteolysis and blocks RANKL/RANK interaction. TNF-α, IL-6 and IL-1 may also play a role in the osteoclastic differentiation and increase production of both RANKL and OPG. These cytokines also act directly on osteoclasts and their precursors and additionally they have an important effect in stimulating RANKL production by osteoblastic cells and in acting synergistically with RANKL (11).

The results of the present study showed that the stromal spindle-shaped cells in CGCGs demonstrated increased TNF-α, IL-6 and decreased IL-1β immunohistochemical expression compared to the PGCGs. Although, immunohistochemical overexpression may not necessarily reflect overproduction of these osteoclastogenic molecules by the spindle cells in CGCGs, a possible alteration in the synthesis or/and activity of these regulatory cytokines within the bone microenvironment may enhance osteolysis through the stimulation of osteoclast progenitor cells, given the fact that intraosseous lesions cause bone resorption. The MGCs in PGCG may occasionally show bone resorptive capacity adjacent to the lesion. The cellular composition in PGCG has been showed to be similar with that in giant cell lesions of different sites and MGCs express the same osteolytic proteases and osteoclastactivating cytokines involved in bone metabolism (30). In PGCG, the TNF-α, IL-6 and IL-1β interrelations may control the cellular activities of the different cell populations (multinuclear cells, monocytes/macrophages, spindle fibroblasts/osteoblasts) contributing possibly mainly in the mechanisms of tumor growth, and occasionally of osteolysis. Friedrich et al. (30) analyzed the expression of proteases relevant for osteolysis in giant cell lesions and showed that despite of the strong cathepsin K expression by the MGCs in PGCGs, there was no radiological evidence for jaw osteolysis adjacent to the examined lesions. According to these authors, the capacity of cathepsin K to degrade bone seems to be related not to the high amounts of this protein in giant cells, but to the topography of the lesion in relation to the adjacent bone, since osteoclasts more distantly located to bone may contain an inactive form (procathepsin K) and not the mature, active cathepsin K. 

In conclusion, the proinflammatory cytokines TNF-α, IL-6 and IL-1β seem to be involved in the growth process of peripheral and central GCGs of the jaws. Further studies are necessary to clarify the functional role of these cytokines in the development of PGCGs and CGCGs and to determine whether control over these proteins may provide another strategy for future medical treatment of these tumors.
